# Cell Reprogramming: Therapeutic Advances and Challenges

**DOI:** 10.34133/research.1472

**Published:** 2026-11-10

**Authors:** Aiqing Li, Zhipeng Wang, Zhaojie Lyu, Zhouting Tuo, Xinrui Li, Rui-Cheng Wu, Jie Wang, Dilinaer Wusiman, Yifan Wang, Qi Zhang, Wei Xiong, Bo Zhou, Dechao Feng

**Affiliations:** ^1^ The Center of Psychosomatic Medicine, Sichuan Provincial People’s Hospital, University of Electronic Science and Technology of China, Chengdu, Sichuan, China.; ^2^ Department of Urology, Sichuan Provincial People’s Hospital, University of Electronic Science and Technology of China, Chengdu, Sichuan, China.; ^3^ Department of Urology, Institute of Precision Medicine, Peking University Shenzhen Hospital, Shenzhen, Guangdong, China.; ^4^ Department of Urology, Tianjin Institute of Urology, The Second Hospital of Tianjin Medical University, Tianjin, China.; ^5^ Urology & Nephrology Center, Department of Urology, Zhejiang Provincial People’s Hospital (Affiliated People’s Hospital), Hangzhou Medical College, Hangzhou, Zhejiang, China.; ^6^ Division of Surgery & Interventional Science, University College London, London, UK.; ^7^ Purdue Institute for Cancer Research, Purdue University, West Lafayette, IN, USA.; ^8^ Sichuan Provincial Center for Mental Health, The Center of Psychosomatic Medicine, Sichuan Academy of Medical Sciences & Sichuan Provincial People’s Hospital, Chengdu, Sichuan, China.; ^9^ Clinical Medicine Research Institute, Zhejiang Provincial People’s Hospital, Hangzhou Medical College, Hangzhou, Zhejiang, China.

## Abstract

Cell reprogramming, which forcibly alters cell fate, offers a novel therapeutic strategy for various refractory diseases. Unlike previous reviews focused on a single disease context or a single reprogramming modality, this review is organized around therapeutic applications. It summarizes advances in direct reprogramming, partial reprogramming, and cell fusion-induced reprogramming and further compares their mechanisms, challenges in clinical translation, and safety boundaries. In oncology, reprogramming cancer cells into antigen-presenting cells elicits durable antitumor immunity and remodels the tumor microenvironment, whereas converting cancer cells into nonmalignant somatic cells can reduce their malignant phenotype and tumorigenic potential. In neurodegenerative diseases, direct reprogramming has converted fibroblasts or astrocytes into functional dopaminergic and cortical neurons both in vitro and in vivo. In mouse models of progeria and natural aging, partial reprogramming strategies markedly extend lifespan, ameliorate multiorgan pathology, and enhance tissue regenerative capacity. However, the strength and reproducibility of these findings vary across experimental models, and reported therapeutic benefits do not necessarily establish complete, stable, or safe cell-fate conversion. Against this predominantly preclinical background, ER-100 is the first regenerative therapy based on partial epigenetic reprogramming to receive Food and Drug Administration Investigational New Drug approval and enter clinical testing. It therefore provides an important reference point for the transition from animal studies to human trials. Cell fusion-induced reprogramming has context-dependent roles in tumor immunity. Fusion between tumor cells and immune cells may promote malignant progression. In contrast, dendritic cell–tumor cell fusion vaccines and chimeric exosomes can activate antitumor immune responses in preclinical models and early clinical studies. Major barriers to clinical translation include low reprogramming efficiency, an incompletely defined safety window, and the need to select in vivo or ex vivo strategies according to disease context. In addition, insufficient delivery efficiency, poor targeting, and long-term tumorigenic risk remain important constraints on clinical application. Looking ahead, single-cell multiomics and artificial intelligence may help optimize reprogramming factor combinations, while nonviral systems may improve targeted delivery. Integration with tissue engineering may further facilitate clinical translation across oncology, aging, regenerative medicine, autoimmune diseases, and neuropsychiatric disorders.

## Introduction

Cell reprogramming refers to the forced alteration of a cell’s epigenetic state and gene-expression network, converting it from one differentiated fate into another cell type with new functions or developmental potential [1]. Lineage plasticity is the ability of a cell to reconfigure its existing identity and enter alternative cell states, and it is an important biological basis of cell reprogramming [2]. Cell reprogramming is not a direct switch from one stable identity to another. Rather, it is a continuous process involving progressively increased lineage plasticity, gradual weakening of the original identity program, and the emergence of intermediate states. This process has both therapeutic value and potential risks: Transient lineage infidelity can promote tissue regeneration during injury repair, whereas in cancer, lineage plasticity can promote intratumoral heterogeneity, therapy resistance, metastatic progression, and immune escape [3]. Because lineage plasticity can both support therapeutic benefit and drive disease progression, artificial cell reprogramming must strike a balance between exploiting plasticity and preventing loss of cell identity control.

Conceptually, cell reprogramming can be understood within a lineage framework of reverse or lateral cell-fate conversion [4,5]. Induced pluripotent stem cells (iPSCs) represent a reverse reset from terminal differentiation to an early developmental pluripotent state. In the iPSC field, the landmark breakthrough came from Takahashi and Yamanaka in 2006 [6], who first showed that 4 transcription factors, OCT4, SOX2, KLF4, and c-MYC (OSKM), can reprogram somatic cells into a pluripotent stem-cell state. Direct reprogramming represents lateral cross-lineage conversion between mature lineages. A foundational milestone in direct lineage reprogramming was the demonstration by Davis in 1987 that the single myogenic regulatory factor MYOD1 could convert C3H10T1/2 fibroblasts into stable myoblasts. The converted cells formed multinucleated, myosin-positive syncytia and expressed multiple skeletal-muscle structural genes [7]. Partial reprogramming achieves functional renewal through limited epigenetic remodeling without compromising the stability of cell identity. Ocampo et al. [8] first showed in vivo that short-term, cyclic expression of OSKM in mice can markedly reverse aging-associated phenotypes and extend lifespan without inducing full pluripotency, thereby clearly introducing the concept of partial reprogramming. Overall, reprogramming methods have expanded rapidly, mechanistic and methodological understanding has deepened [9], and these approaches have been widely applied in developmental biology, disease modeling, drug discovery, cell therapy, and cancer immunotherapy [1].

In recent years, cell reprogramming has shown substantial potential for treating multiple refractory diseases. iPSC technology can reprogram somatic cells into a pluripotent state and then induce their differentiation into target cells for transplantation therapy [10]. This strategy has made important progress in models of limbal stem-cell deficiency [11], Parkinson’s disease (PD) [12], diabetes [13], and cancer [14]. Because the clinical applications of iPSC technology have been systematically reviewed elsewhere [1,9,15], this review does not revisit them in detail and uses them only as a reference point for clinical translation. Direct reprogramming has shown considerable promise in myocardial infarction, neural injury, and cancer therapy [1]. Partial reprogramming, as an emerging strategy, offers new ideas for treating aging and degenerative diseases and for regenerative medicine [16].

Cell fusion is a special form of cell reprogramming. Cell fusion involves membrane fusion, cytoplasmic mixing, and the formation of heterokaryons or synkaryons. This process allows trans-acting factors, cytoplasmic components, and epigenetic regulatory environments from one parent cell to influence the nucleus of another. Some fusion events can thereby trigger stable transcriptional, epigenetic, and functional remodeling, enabling the fusion product to acquire a cell state distinct from either parent. Therefore, not all fusion events cause a change in cell identity; in this review, events that generate stable reconstruction of cell state are defined as cell fusion-induced reprogramming [17,18]. The inclusion of cell fusion-induced reprogramming in the clinical application framework of this review is based on its dual pathological and therapeutic significance [19]. On the one hand, fusion between tumor cells and mesenchymal stem cells or immune cells can cause genomic mixing and chromosomal instability, thereby driving malignant heterogeneity, metastasis, and drug resistance [19], and it is substantially associated with poor prognosis in several cancers [20–25]. On the other hand, dendritic cell–tumor cell fusion vaccines and hybrid cell-derived exosome strategies have shown potential for activating antitumor immunity in clinical trials and animal models [26–28]. This duality makes cell fusion both a valuable therapeutic target and an important biological tool.

Unlike previous reviews that have largely focused on a single disease type, such as aging [29], neurodegenerative diseases [30], regenerative medicine [9], or malignancy [31], this review takes disease-oriented therapeutic applications as its main organizing framework. The main innovations are as follows: (a) It systematically summarizes recent progress of 3 reprogramming strategies across major disease areas. (b) It compares these strategies across key translational dimensions, including cell-identity reconstruction, epigenetic barriers, intermediate-state stability, delivery efficiency, and safety profiles. (c) It incorporates cell fusion-induced reprogramming as a distinct modality with both pathological and therapeutic significance, focusing on its potential, limitations, and safety boundaries in cancer immunotherapy. (d) It proposes an operational framework for defining the safe therapeutic window. Based on this framework, this review shifts the discussion beyond the proof-of-concept question of whether cell fate can be changed. It instead focuses on whether such changes are stable, reproducible, and safe, and how appropriate strategies can be selected to achieve safe and controllable clinical translation.

## Cell Reprogramming Technologies

### Direct reprogramming

Direct reprogramming, also known as transdifferentiation, changes a somatic cell from one lineage to another without an intermediate pluripotent state [5]. This approach bypasses the iPSC intermediate stage and directly generates functional terminal cells. In theory, it is faster and safer because it avoids the tumor risk linked to pluripotency [32]. Current direct reprogramming methods mainly rely on transcription factor overexpression or chemical small-molecule induction [1]. Transcription factor overexpression forces activation of key regulatory genes of the target lineage and drives global conversion of cell identity. Chemical small-molecule strategies alter cell fate without introducing exogenous genes by targeting epigenetic-modifying enzymes, signaling pathways, or metabolic nodes and therefore have advantages in safety and clinical controllability. Additionally, some studies have combined transcription factors or small molecules, or incorporated miRNAs, to improve efficiency. At the level of cell-type conversion, this technology has been reported to generate multiple functional terminal cell types, including neurons, cardiomyocytes, hepatocytes, pancreatic beta-like cells, and chondrocytes, both in vitro and in vivo (technical methods summarized in Table 1 [33–59]).

**Table 1.** Representative direct reprogramming strategies across disease applications

Disease application category	Starting cell type	Key factors	Delivery method	Terminal cell type	References
Malignancy	Human leukemic blasts	CEBPα, PU.1	Nucleofection	Macrophages	33
	Human melanoma cells	PU.1, IRF8, BATF3	Adenovirus-mediated delivery	cDC1	34
	Mouse/human fibroblasts	PU.1, IRF8, BATF3	Lentiviral vector	cDC1	35
	Effector T cells	LEF1, KLF7, ID3, EOMES, BCL6, TCF7, FOXP1, FOXO1	mRNA electroporation	Stem-like memory CD8^+^ T cells	36
	Human squamous cell carcinoma cells	MITF, LEF1, SOX9, SOX10	Doxycycline-inducible lentiviral vector	Melanocyte-like cells	37
	Hepatocellular carcinoma cells	HNF1A, HNF4A, FOXA3	Adenoviral vector	Hepatocytes	38
	Primary mouse pro-B leukemia cells	Constitutively active NOTCH1	Retrovirus	Thy1.2+CD19- T-lineage cells	39
	Invasive breast cancer cells	PPARγ agonist, MEK inhibitor	Chemical induction	Functional adipocytes	40
Neurodegenerative diseases	Human fibroblasts	BRN2, ASCL1, MYT1L	Lentiviral infection	Functional neurons	41
	Human skin fibroblasts	ASCL1, NR4A2, LMX1A	Doxycycline-inducible lentivirus	Dopaminergic neurons	42
	Human fibroblasts	miR-9/9*, miR-124, ASCL1, MYT1L, NEUROD2	Lentiviral vector	Mature neurons	43
	Skin fibroblasts	miR-9/9*, miR-124, NEUROD2, MYT1L	Lentiviral transduction	Cortical neurons	44
	Astrocytes	NEUROD1	AAV vector	Functional neurons	45
	Human fibroblasts	Valproic acid, CHIR99021, RepSox, Forskolin, SP600125, GO6983, Y-27632	Chemical induction	Glutamatergic neurons	46
	Human fetal astrocytes	DAPT, CHIR99021, SB431542, LDN193189	Chemical induction	Glutamatergic neurons	47
Regenerative medicine, cardiovascular	Primary HCFs; human skin fibroblasts	MEF2C, GATA4, TBX5, TBX20, miR-133	Lentiviral transduction	Cardiomyocytes	48
	Human fibroblasts	miR-1, miR-133, miR-208, miR-499, JAK inhibitor I	Liposomal transfection/intramyocardial lentiviral injection	Cardiomyocyte-like cells	49
	HCFs	MYOCD, ASCL1, miR-133	AAV vector	Cardiomyocyte-like cells	50
	Human fibroblasts	CHIR99021, A83-01, BIX01294, AS8351, SC1, Y27632, OAC2, SU16F, JNJ10198409	Chemical induction	Cardiomyocyte-like cells	51
	HCFs	GATA4, MEF2C, TBX5, MYOCD, SB431542, XAV939	Retrovirus	Induced cardiomyocyte-like cells	52
Regenerative medicine, liver	Human fetal fibroblasts, adult fibroblasts, and adult adipose-derived MSCs	FOXA3, HNF1A, HNF4A	Lentivirus	Functionally mature human hepatocytes	53
	Human embryonic fibroblasts	HNF1A, HNF4A, HNF6, ATF5, PROX1, CEBPA	Lentivirus	Human hepatocytes	54
Regenerative medicine, endocrine	Human fibroblasts	OCT4, SOX2, KLF4, anti-p53 shRNA	Episomal plasmid electroporation	Pancreatic beta-like cells	55
	Mouse embryonic fibroblasts	VPA, CHIR98014, RepSox, TTNPB, celecoxib	Chemical induction	Functional chondrocytes	56
	Fibroblasts	Forskolin, 20α-hydroxycholesterol, luteinizing hormone, SB431542	Chemical induction	Functional Leydig cells	57
Regenerative medicine, visual system	Fibroblasts	VPA, CHIR99021, RepSox, Forskolin, IWR1	Chemical induction	Photoreceptor-like cells	58
Autoimmune system	Proinflammatory human Th17 cells	Aminooxyacetic acid	Chemical induction	Regulatory iTreg cells	59

AAV, adeno-associated virus; ASCL1, achaete-scute family bHLH transcription factor 1; ATF5, activating transcription factor 5; BATF3, basic leucine zipper ATF-like transcription factor 3; BCL6, BCL6 transcription repressor; BRN2, POU class 3 homeobox 2; C/EBPα, CCAAT/enhancer-binding protein alpha; cDC1, conventional type 1 dendritic cell; CD, cluster of differentiation; EOMES, eomesodermin; FOXA3, forkhead box A3; FOXP1, forkhead box P1; FOXO1, forkhead box O1; GATA4, GATA-binding protein 4; HCFs, human cardiac fibroblasts; HNF1A, hepatocyte nuclear factor 1 alpha; HNF4A, hepatocyte nuclear factor 4 alpha; HNF6, hepatocyte nuclear factor 6; ID3, inhibitor of DNA binding 3; IRF8, interferon regulatory factor 8; iTreg cells, induced regulatory T cells; JAK, Janus kinase; KLF7, Krüppel-like factor 7; LEF1, lymphoid enhancer-binding factor 1; LMX1A, LIM homeobox transcription factor 1 alpha; MEF2C, myocyte enhancer factor 2C; MEK, mitogen-activated protein kinase kinase; miR, microRNA; MITF, microphthalmia-associated transcription factor; mRNA, messenger RNA; MSCs, mesenchymal stem cells; MYOCD, myocardin; MYT1L, myelin transcription factor 1-like; NEUROD1, neuronal differentiation 1; NEUROD2, neuronal differentiation 2; NOTCH1, notch receptor 1; NR4A2, nuclear receptor subfamily 4 group A member 2; OCT4, octamer-binding transcription factor 4; PPARγ, peroxisome proliferator-activated receptor gamma; PROX1, prospero homeobox 1; PU.1, transcription factor PU.1 (encoded by SPI1); shRNA, short hairpin RNA; SOX2, SRY-box transcription factor 2; SOX9, SRY-box transcription factor 9; SOX10, SRY-box transcription factor 10; TBX5, T-box transcription factor 5; TBX20, T-box transcription factor 20; TCF7, transcription factor 7; Th17 cells, T helper 17 cells; VPA, valproic acid

### Partial reprogramming

Partial reprogramming uses transient and controlled reprogramming factor expression to reduce aging-associated cellular features (such as DNA damage and telomere shortening) [29]. Its goal is to reset the epigenetic and functional states of aged cells while avoiding acquisition of pluripotency and the associated tumorigenic risk [60]. Its success depends on precise spatiotemporal control of reprogramming factor expression [8]. At present, partial reprogramming is mainly achieved through 2 routes: short-term treatment with small-molecule combinations and transient transgene expression. Small-molecule strategies use chemical cocktails to treat aged cells briefly and can improve multiple aging-associated phenotypes without introducing genetic material [61]. Transient transgene strategies use cyclic doxycycline induction, transient mRNA transfection, or adeno-associated virus (AAV)-mediated gene delivery to achieve time-limited expression of reprogramming factors [8,62,63]. In addition, expression systems driven by senescence-responsive promoters, such as the p16 promoter, can restrict reprogramming factors to senescent cell populations and further improve targeting [63]. This strategy has achieved proof-of-concept progress in reversing multiorgan aging phenotypes, promoting tissue regeneration, and repairing specific neural functions (technical methods summarized in Table 2 [8,61–66]).

**Table 2.** Representative partial reprogramming strategies

Disease application category	Starting cell type	Key factors	Delivery method	References
Aging-related diseases	Aged human fibroblasts	CHIR99021, DZNep, Forskolin, TTNPB, Valproic acid, Repsox, and Tranylcypromine	Chemical induction	61
	Multiple cell types from progeroid/aged wild-type mice	OCT4, SOX2, KLF4, c-MYC	Cyclic doxycycline induction	8
	Multiple naturally aged human cell types	OCT4, SOX2, KLF4, c-MYC, LIN28, NANOG	Transient nonintegrating mRNA expression	62
	Multiple cell types from progeroid/naturally aged mice	OCT4, SOX2, KLF4	AAV-DJ viral vector	63
Regenerative medicine, visual system	Retinal ganglion cells	OCT4, SOX2, KLF4	AAV viral vector	64
Regenerative medicine, intervertebral disc	Aged nucleus pulposus cells	OCT4, SOX2, KLF4	Engineered exosomes	65
Regenerative medicine, peripheral nerve	Schwann cells	OCT4, SOX2, KLF4, c-MYC	Doxycycline induction	66

AAV, adeno-associated virus; AAV-DJ, engineered hybrid AAV serotype; c-MYC, cellular MYC proto-oncogene protein; KLF4, Krüppel-like factor 4; LIN28, lin-28 homolog A; mRNA, messenger RNA; NANOG, Nanog homeobox; OCT4, octamer-binding transcription factor 4; OSK, OCT4/SOX2/KLF4; OSKM, OCT4/SOX2/KLF4/c-MYC; SOX2, SRY-box transcription factor 2

### Cell fusion-induced reprogramming

Cell fusion refers to the formation of hybrid cells containing the nuclei of both parent cells or fused nuclei through membrane fusion and cytoplasmic mixing between 2 cells [16]. Unlike direct or partial reprogramming, which usually rely on transgenes or chemical induction, cell fusion-induced reprogramming changes cell state through physical cell merging rather than simple lineage conversion [16]. It can be induced by chemical fusogens, such as polyethylene glycol, or by electrofusion [67]. Depending on the fusion partners, the resulting hybrid cells may acquire pluripotency, metastatic traits, or immunomodulatory functions.

The dendritic cell–tumor cell fusion vaccine is the most extensively studied cell-fusion-based therapeutic strategy. It involves fusing patient-derived dendritic cells with autologous tumor cells using chemical fusogens such as polyethylene glycol or electrofusion [68]. Another approach uses macrophages that engulf tumor cell nuclei to form hybrid cells; the chimeric exosomes secreted by these hybrids have both lymph node-targeting and tumor microenvironment (TME)-modulating functions [69]. However, the efficiency of fusion and fusion-induced reprogramming remains low. Embryonic factors such as NANOG or SALL4, activation of Wnt/β-catenin or Akt signaling, and tissue injury or inflammatory conditions can enhance cell fusion-induced reprogramming. In models of muscle, brain, and liver injury, fusion between bone marrow-derived cells and injured tissue cells increases and may contribute to tissue repair [18].

The biological consequences of cell fusion-induced reprogramming are highly context-dependent. When tumor cells spontaneously fuse with stromal or immune cells, the resulting hybrids may promote malignant progression if they survive and acquire proliferative, migratory, invasive, or immune-evasive capacities [70,71]. Tumor-macrophage and tumor-leukocyte hybrids can carry both malignant and hematopoietic features. These hybrids have been linked to tumor heterogeneity, progression, and poor prognosis [20]. Thus, the protumorigenic effect of cell fusion depends on the traits acquired by the fusion products and their selection in vivo.

By contrast, dendritic cell–tumor cell fusion vaccines are designed to direct the outcome of fusion toward immune activation. In this strategy, tumor cells provide a broad repertoire of tumor antigens, while dendritic cells provide antigen-processing capacity, major histocompatibility complex (MHC) molecules, and costimulatory signals required for T-cell activation [68]. Unlike spontaneous fusion in vivo, these fusion products are usually generated and inactivated under controlled ex vivo conditions, allowing fusion efficiency, antigen-presenting phenotype, residual unfused tumor cells, and product safety to be evaluated before reinfusion.

### Shared mechanisms and differences among the 3 reprogramming strategies

After clarifying the differences in induction routes among these 3 strategies, it is necessary to further examine their mechanistic commonalities and distinctions. Although direct reprogramming, partial reprogramming, and cell fusion-induced reprogramming differ in induction mode and kinetic trajectory, all 3 fundamentally depend on reconstruction of cell identity regulatory networks and systematic remodeling of epigenetic states [72]. Their shared mechanistic basis includes rearrangement of chromatin accessibility, coordinated changes in DNA methylation and histone modifications, and reconfiguration of enhancer networks. Different transcription factor combinations show a degree of functional modularity. Most effective reprogramming systems involve 3 functional classes of factors. These include factors that promote chromatin opening, core factors that establish or maintain lineage transcriptional programs, and auxiliary factors that enhance cell survival or conversion efficiency [73,74].

Different reprogramming strategies differ in the depth of lineage conversion and the scope of epigenetic remodeling. Direct reprogramming usually requires sustained activation of the target-lineage regulatory network to achieve stable identity conversion [5]. Partial reprogramming induces only limited epigenetic resetting while avoiding full entry into pluripotency [29]. Cell fusion-induced reprogramming reconfigures cell identity programs through competition and dominance among transcriptional regulatory networks in heterokaryons, rather than simple genomic homogenization [17]. These mechanistic differences jointly determine the kinetic features of each strategy, including enhancer state and cell-cycle dependence, and may further influence their potential tumorigenic risk profiles (mechanistic features summarized in Table 3 [8,17,51,64,75–102]).

**Table 3.** Mechanistic features of direct reprogramming, partial reprogramming, and cell fusion-induced reprogramming

Mechanistic feature	Direct reprogramming	Partial reprogramming	Cell fusion-induced reprogramming
Chromatin accessibility	Chromatin opening is critical for initiating direct reprogramming and lineage conversion. Fibroblasts show changes within 12 h after factor expression [75], and single-cell ATAC-seq also confirms accessibility remodeling during early cardiac reprogramming [76].	Partial reprogramming can remodel chromatin accessibility early in aged cells, including closure of AP-1-associated regions, down-regulation of mesenchymal programs, and restoration of a youthful cell identity [77].	Chromatin is rapidly remodeled after fusion; pioneer factors such as OCT4 and NANOG promote opening of somatic-cell chromatin [78,79].
DNA methylation	Direct reprogramming is accompanied by lineage-specific DNA methylation remodeling, but usually retains some donor age and lineage memory. Extensive remodeling occurs in both cardiac and neuronal reprogramming [80,81], and promoting demethylation can improve neuronal conversion efficiency [82].	DNA methylation remodeling is an important mechanism of rejuvenation during partial reprogramming. OSK can restore youthful methylation features and reduce epigenetic age [64,83].	In cell fusion-induced reprogramming, DNA replication drives methylation remodeling [84].
Enhancer reprogramming	Direct reprogramming requires closure of donor enhancers and establishment of target-lineage enhancers; incomplete remodeling is often associated with failure. Cardiac factors can cooperatively activate stage-specific enhancers, promoting establishment and maturation of the target program [85,86].	Partial reprogramming can restore youthful enhancer states in aged tissues [87].	Early after fusion, OCT4, SOX2, NANOG, and related factors can bind enhancers of pluripotency genes and initiate core transcriptional networks [88].
Cell-cycle dependence	Cell-cycle progression is not universally required; its role depends on the starting cell, target lineage, and induction protocol. Neuronal conversion can occur in nonproliferating cells, whereas cardiac reprogramming is often inhibited by active proliferation, and cell-cycle inhibition or synchronization can improve efficiency [89].	Partial reprogramming has no universal cell-cycle dependence. Proliferation may participate in some tissue responses, but it is not required for rejuvenation or identity change [64,90].	Highly cell-cycle dependent: Embryonic stem cell-induced S-phase entry and DNA replication are required for fusion-mediated reprogramming [91].
H3K9me3	H3K9me3 is an important heterochromatin barrier to direct reprogramming. Reducing its deposition or inhibiting related enzymes can improve cardiac and hepatocyte reprogramming [51,92], although premature removal may inhibit neuronal formation [93].	Direct evidence is limited; existing studies suggest that cyclic OSKM can alter H3K9me3 levels in specific tissues, but the causal relationship among these changes, rejuvenation, identity maintenance, and functional improvement remains to be clarified [94].	Direct evidence is lacking.
H3K27me3	H3K27me3 is closely associated with transcriptional repression. During direct cardiac reprogramming, target-gene promoters lose H3K27me3, whereas fibroblast genes gradually acquire this mark [80]. Promoting H3K27 demethylation can relieve repression of target genes and improve cardiomyocyte reprogramming efficiency [95,96].	Direct evidence is lacking.	Direct evidence is lacking.
Intermediate-state stability	Intermediate states, branched trajectories, and alternative fates exist, but their long-term stability has not been demonstrated. Fate divergence often forms early, and failed cells may enter other lineages or return to the original state [97,98].	Progenitor-like, fetal-like, or transcriptional intermediate states can form, but they are usually transient, reversible, and unstable; after OSKM withdrawal, cells typically return to their original identity [99].	Transient heterokaryons and bifurcating intermediate states exist, and the overall state is unstable [17].
Tumorigenic risk	Risk mainly arises from viral or DNA vector integration and use of oncogenes. Nonintegrating modified mRNA or small-molecule approaches may theoretically reduce insertional mutagenesis and tumorigenic risk [100].	Risk is influenced by OSKM dose, duration, delivery method, genetic background, and tissue type. Transient or cyclic expression can reduce but not eliminate teratoma risk; removing MYC also does not guarantee safety, and long-term safety in humans remains unclear [8,101].	Polyploidy and karyotypic instability indicate potential tumorigenic risk [102].

AP-1, activator protein 1; ATAC-seq, assay for transposase-accessible chromatin using sequencing; c-MYC, cellular MYC proto-oncogene protein; H3K9me3, trimethylation of histone H3 lysine 9; H3K27me3, trimethylation of histone H3 lysine 27; KLF4, Krüppel-like factor 4; mRNA, messenger RNA; NANOG, Nanog homeobox; OCT4, octamer-binding transcription factor 4; OSK, OCT4/SOX2/KLF4; OSKM, OCT4/SOX2/KLF4/c-MYC; SOX2, SRY-box transcription factor 2

Beyond the mechanistic differences described above, the representative protocols in Tables 1 and 2 provide practical starting points but require strategy-specific optimization. Each strategy requires distinct optimization. Transcription factor-mediated reprogramming requires control of factor expression, delivery efficiency, and withdrawal timing. Chemical reprogramming requires optimization of compound combinations, doses, treatment sequence, and duration, whereas cell fusion requires optimization of fusion partners, fusion conditions, and product purification. Regardless of the strategy, protocol evaluation should extend beyond conversion efficiency or marker expression to include target-cell identity, functional maturation, postinduction stability, and abnormal proliferation or tumorigenic potential.

## Therapeutic Advances

### Therapeutic advances in malignancies

Although surgery, radiotherapy, immunotherapy, and targeted therapy are the mainstays of cancer treatment [103–105], their efficacy is limited by tumor stage, TME heterogeneity, and immune evasion, ultimately leading to a substantial disease burden [106,107]. Cancer cells possess genetic and epigenetic plasticity, offering the possibility to “reverse their fate” through reprogramming [108,109]. Current strategies for reprogramming cancer cells mainly involve either converting them into immune-like cells to activate antitumor immunity or transforming them into nonmalignant somatic-like cells (Fig. 1). In addition, dendritic cell–tumor cell fusion vaccines and chimeric exosomes derived from cell fusion have opened new avenues for cancer immunotherapy.

**Fig. 1.**
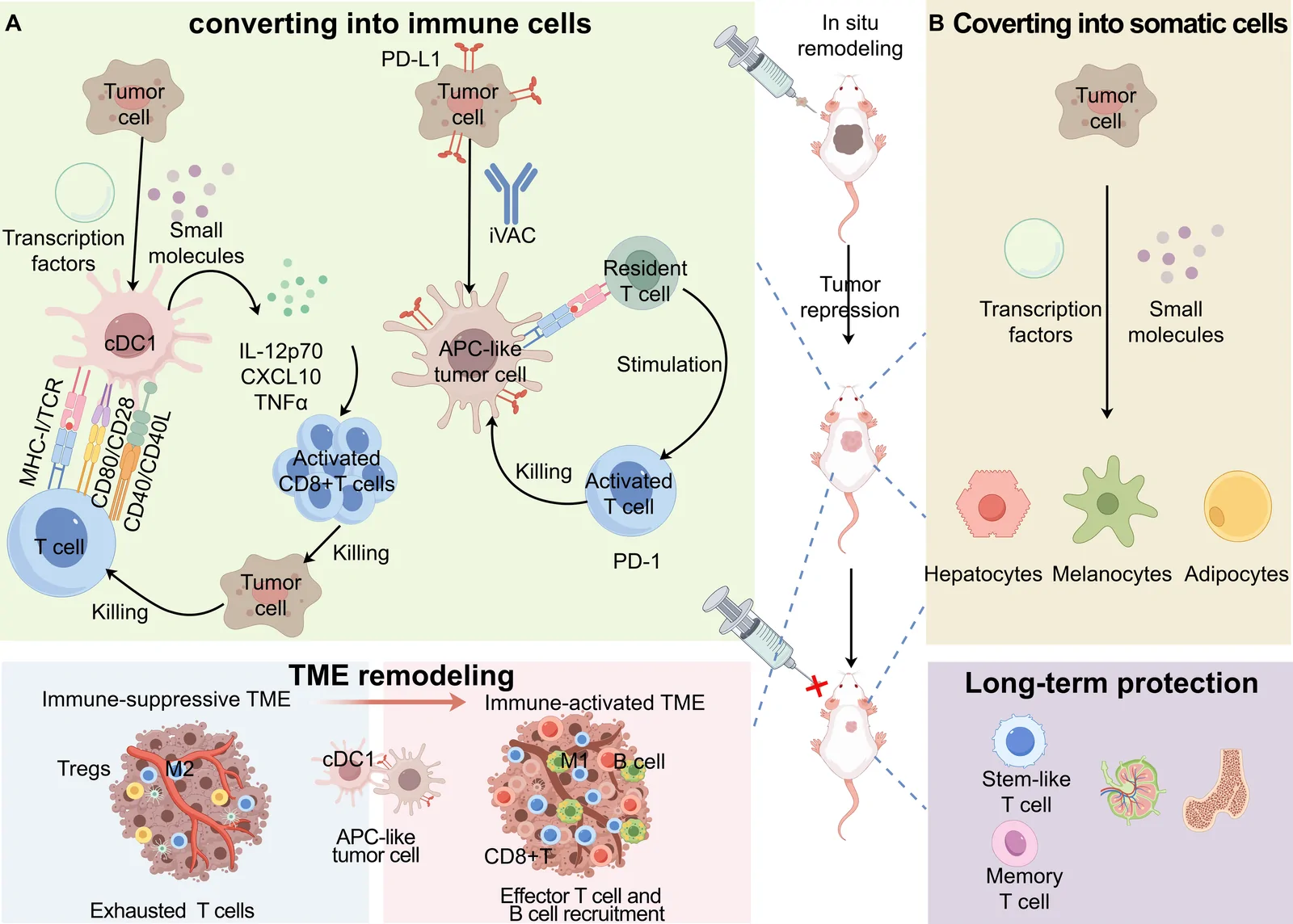
Cell reprogramming intervention strategies in cancer therapy. (A) Reprogramming cancer cells into immune-like cells. Transcription factors, small molecules, or the intratumoral vaccination chimera (iVAC) can convert cancer cells into conventional type 1 dendritic cells (cDC1s) or antigen-presenting cell (APC)-like cells. These reprogrammed cells enhance antigen presentation, activate CD8^+^ T-cell responses, and remodel the immunosuppressive tumor microenvironment into an immune-activated state. The resulting expansion of effector and memory T-cell populations may support tumor regression and durable antitumor immune protection; (B) Reprogramming cancer cells into nonmalignant somatic-like cells. Transcription factors or small molecules can redirect cancer cells toward hepatocyte-, melanocyte-, or adipocyte-like states, thereby suppressing proliferation, invasion, metastasis, and tumorigenic potential. However, phenotypic conversion does not necessarily eliminate the original oncogenic alterations, and long-term stability and safety require further evaluation. CD, cluster of differentiation; CD40L, CD40 ligand; CXCL10, C-X-C motif chemokine ligand 10; IL-12p70, interleukin-12 p70; M1, classically activated macrophage; M2, alternatively activated macrophage; MHC-I, major histocompatibility complex class I; PD-1, programmed cell death protein 1; PD-L1, programmed death-ligand 1; TCR, T-cell receptor; TME, tumor microenvironment; TNF-α, tumor necrosis factor alpha; Tregs, regulatory T cells.

Reprogramming cancer cells toward functional antigen-presenting cells may remodel the TME and enhance antitumor immune responses. In preclinical models, such approaches have increased tumor immunogenicity, promoted T-cell activation, and, in some settings, generated immune responses detectable after tumor rechallenge. For example, C/EBPα and PU.1 coexpression reprograms human leukemic blasts into nontumorigenic macrophages that fail to establish disease after transplantation [33]. Another study reprogrammed human and mouse cancer cell lines from hematologic and solid tumors into conventional type 1 dendritic cells (cDC1s) with antigen-presenting capacity. The resulting cells up-regulated surface expression of MHC class I molecules and costimulatory molecules, such as CD40, CD80, and CD86 [110], thereby increasing immunogenicity. They were also able to uptake and cross-present exogenous antigens and secrete multiple proinflammatory cytokines (e.g., IL-12p70, CXCL10, and tumor necrosis factor alpha), effectively activating CD8^+^ T cells, inducing their proliferation and specific killing of tumor cells. Some mice achieved complete tumor regression [110].

The above studies mainly evaluated immune function after ex vivo cancer cell fate conversion, whereas subsequent work attempted to implement reprogramming directly within tumors. One study directly reprogrammed tumor cells into cDC1-like cells in vivo, demonstrating preclinical efficacy in multiple mouse melanoma models (including B16 and YUMM1.7). When administered alone, the monotherapy induced either complete tumor regression or a marked extension of median survival. Its combination with immune checkpoint inhibitors (anti-programmed cell death protein 1 [PD-1]/cytotoxic T-lymphocyte associated protein 4) led to even higher rates of complete remission. This treatment also remodeled the TME. It promoted tertiary lymphoid structure formation and increased the infiltration of CD8^+^ T cells, B cells, and other immune cells. At the same time, it reduced exhausted T cells and immunosuppressive regulatory T cells. Most importantly, it substantially increased the levels of effector memory T cells and stem-like T cells, which are responsible for long-term immune surveillance, thereby establishing durable antitumor immune memory that enabled cured mice to resist tumor rechallenge [34]. Another study overexpressed STX11 to convert autologous tumor cells into dendritic cell-like cells, then engineered the cell membranes of these modified cells to produce artificial dendritic cell-derived vesicles, constructing a personalized nanovaccine. In a postsurgical metastatic mouse model, the monotherapy inhibition rate was approximately 57% to 76%, and combination with anti-programmed death-ligand 1 (PD-L1) achieved 100% survival [111].

Beyond traditional small-molecule-based reprogramming strategies, another study developed a bifunctional macromolecule termed intratumoral vaccination chimera (iVAC). iVAC converts tumor cells into antigen-presenting cell-like cells, promotes PD-L1 degradation, and enables cross-presentation of exogenous antigens. PD-L1 degradation relieves immunosuppression, and the exogenous antigens activate otherwise nontumor-specific “bystander” T cells in the TME, redirecting them to recognize and kill tumor cells. Activated T cells secrete cytokines such as interferon gamma and tumor necrosis factor, up-regulate granzyme B and perforin, and enhance cytotoxic activity. At the same time, iVAC remodels the TME by increasing proinflammatory M1-type macrophages, decreasing anti-inflammatory M2-type macrophages, and promoting the maturation of cDC1s, thereby synergistically driving a durable and systemic antitumor immune response. The overall response rate was 61% across 28 patient-derived tumor models, reaching as high as 69% in specific human leukocyte antigen subtypes [112].

An alternative approach involves the direct conversion of cancer cells into functionally mature, nonproliferative somatic cells. Reprogramming squamous cell carcinoma cells into melanocyte-like cells markedly reduced their proliferative capacity, metabolic activity, migratory ability, and invasive potential in vitro, and markedly reduced tumorigenic potential in immunodeficient mice over a period of 30 weeks [37]. However, reduced or undetectable tumor formation during a finite xenograft observation period does not establish irreversible elimination of malignant potential, particularly when the original oncogenic genome is retained. In another study, adenoviral delivery of hepatocyte nuclear factors into hepatocellular carcinoma cell lines (HCCLM3 and Huh-7) generated reprogrammed hepatocyte-like cells. These cells exhibited hepatocyte morphology and restored hepatic functions in vitro. Intratumoral injection of the transcription factor combination significantly suppressed xenograft tumor growth and prolonged mouse survival [38]. Notably, lineage choice determines success or failure. In a mouse pro-B leukemia model, conversion to T cells retained the malignant phenotype, whereas differentiation toward the myeloid lineage completely abrogated tumorigenic capacity [39]. Chemical small-molecule reprogramming strategies are also noteworthy. Combining a peroxisome proliferator-activated receptor gamma agonist with a mitogen-activated protein kinase kinase inhibitor converted invasive breast cancer cells into functional adipocytes in multiple mouse breast cancer models (including 4T1, MDA-MB-231 LM2, and patient-derived xenograft models), markedly suppressing primary tumor invasion and lung metastasis [40]. These results indicate that phenotypic improvement of cancer cells does not necessarily mean that genetic abnormalities have been eliminated. Whether the target lineage can stably suppress the original malignant program is a key determinant of therapeutic efficacy.

Dendritic cell–tumor cell fusion vaccines have demonstrated safety and immunogenicity in several early-stage clinical trials. In acute myeloid leukemia, a single-arm phase I trial (NCT03679650) reported a median overall survival of 50 months in vaccinated patients, with 70% remaining relapse-free long-term [26]. A trial in elderly patients after chemotherapy-induced remission (NCT03059485, *n* = 41) showed that the vaccine group had superior 2-year progression-free survival and overall survival compared to controls (36% vs. 23%; 73% vs. 61%) [113], although the statistical significance of these differences was not reported. A phase II trial in multiple myeloma (NCT02728102) did not markedly improve complete remission rates but successfully induced myeloma-specific T-cell clonal expansion [114]. These findings suggest that fusion vaccines can often induce tumor-specific immunity, but immunological responses do not necessarily translate into substantial long-term clinical benefit. Their efficacy may be limited by the immunosuppressive residual TME, high-risk disease biology, effects of combination therapies on immune dynamics, and insufficient sample size and follow-up [114]. To improve effector T-cell function and the persistence of immune responses, current studies are beginning to combine fusion vaccines with newer immunotherapies. A phase I trial in relapsed or refractory multiple myeloma plans to enroll approximately 25 patients (NCT06799026). The study will evaluate the safety, immunological responses, and preliminary antitumor efficacy of an autologous dendritic cell–myeloma cell fusion vaccine combined with the B-cell maturation antigen/CD3 bispecific antibody elranatamab, with or without granulocyte-macrophage colony-stimulating factor [115]. Another phase I trial plans to enroll approximately 25 patients with relapsed or refractory multiple myeloma (NCT07377435). It will evaluate the safety and preliminary efficacy of combining an autologous dendritic cell–myeloma cell fusion vaccine with B-cell maturation antigen chimeric antigen receptor T-cell therapy, including its potential to enhance immune persistence and reduce relapse risk [116]. However, application of this technology in solid tumors faces greater challenges. A phase I/II study in renal cell carcinoma (NCT01441765) was terminated because of failure to recruit participants [117]. A phase I/II melanoma study (NCT00626860) has been completed, but detailed results have not yet been published [118]. Thus, current clinical evidence mainly demonstrates the feasibility and immunogenicity of fusion vaccine production; however, it remains insufficient to confirm a stable survival benefit, and larger controlled studies are still needed (Table 4).

**Table 4.** Clinical trials of dendritic cell–tumor cell fusion vaccines in cancer therapy

Disease type	Clinical trial identifier	Phase	Results	References
AML	NCT03679650	Phase I	Median overall survival of vaccinated patients was 50 months; 70% remained relapse-free long term	26
AML	NCT03059485	Phase II	The vaccine group had better 2-year PFS (36% vs. 23%) and OS (73% vs. 61%) than controls	113
MM	NCT02728102	Phase II	Did not markedly improve complete response rate, but induced clonal expansion of myeloma-specific T cells	114
RCC	NCT01441765	Phase I/II	Terminated because of failure to recruit participants	117
Melanoma	NCT00626860	Phase I/II	Completed, but detailed results have not been published	118
Relapsed/refractory MM	NCT06799026	Phase I	Recruiting	115
Relapsed/refractory MM	NCT07377435	Phase I	Recruiting	116

AML, acute myeloid leukemia; MM, multiple myeloma; OS, overall survival; PFS, progression-free survival; RCC, renal cell carcinoma

Beyond traditional fusion cell vaccines, studies have exploited exosomes generated from cell fusion to achieve immune regulation. One study used polyethylene glycol to fuse dendritic cells with tumor cells and extracted chimeric exosomes, which markedly suppressed tumor growth in a glioma model and, when combined with anti-PD-1, almost completely eliminated postoperative recurrence [27]. Another study used macrophages to engulf tumor cell nuclei, forming hybrid cells; the secreted chimeric exosomes had both lymph node-targeting and TME-modulating functions, markedly inhibiting primary tumor growth and metastasis in lymphoma, breast cancer, and melanoma models and synergizing with anti-PD-1 to suppress postoperative recurrence [69]. Cell fusion may also generate hybrid cells with protumor functions, making these cells themselves potential therapeutic targets. For example, targeting immunosuppressive molecules expressed on them has been proposed [119]. In a mouse model of anaplastic thyroid cancer, a subpopulation of double-positive VSIG4 and SPP1 tumor-associated macrophages was identified; VSIG4 knockout or antibody blockade combined with SPP1 inhibition showed synergistic antitumor effects [28].

### Aging and related diseases

Current anti-aging strategies mainly focus on clearing senescent cells, modulating metabolism, and inducing autophagy/mitophagy. In recent years, partial reprogramming has been investigated as another approach to aging intervention. Through transient and controllable expression of reprogramming factors, this strategy can attenuate several cellular hallmarks of aging, including DNA damage, epigenetic alterations, and telomere shortening, while preserving cell identity [120].

In aging and neurodegenerative diseases, cell reprogramming is mainly being explored through 2 complementary routes: partial reprogramming to restore youthful cellular states and direct neuronal reprogramming to generate disease-relevant neurons (Fig. 2). In vitro, short-term treatment of aged human fibroblasts with a small-molecule cocktail improved aging-related phenotypes, and the same treatment extended the median lifespan of *C. elegans* by 42.1% [61]. Transient mRNA expression of reprogramming factors also reversed the epigenetic clock in multiple naturally aged human cell types, reduced inflammatory cytokine secretion (such as interleukin-18 [IL-18], IL-1A, GROA, IL-22, IL-8, IL-9, etc.), and restored several regenerative features of aged muscle stem cells [62].

**Fig. 2.**
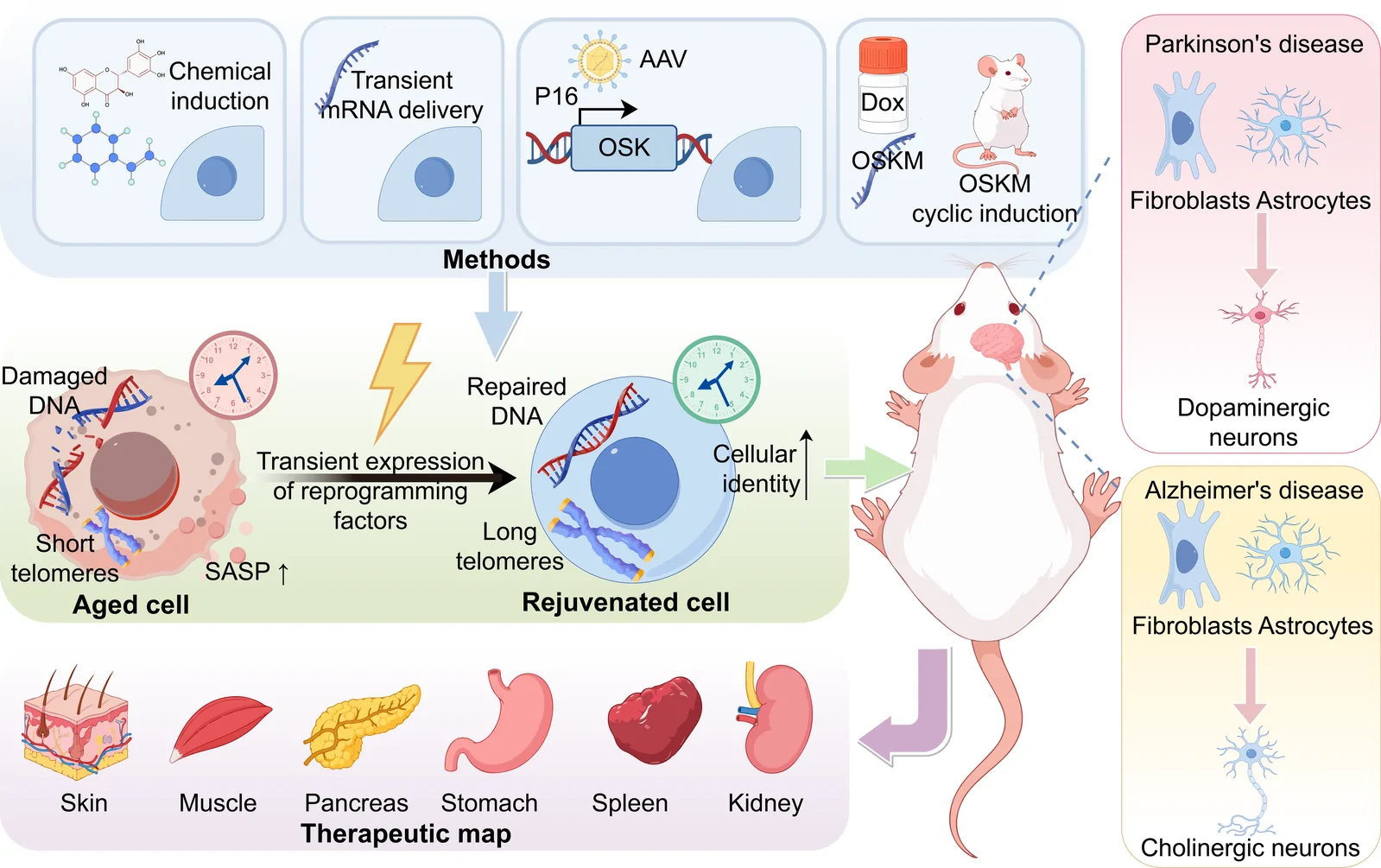
Cell reprogramming strategies for aging and neurodegenerative diseases. Partial reprogramming uses transient small-molecule treatment or controlled expression of reprogramming factors to attenuate aging-associated cellular changes while preserving cell identity. In vivo, it can improve tissue homeostasis and regenerative capacity in multiple organs. Direct neuronal reprogramming converts fibroblasts or astrocytes into disease-relevant neuronal cell types, including dopaminergic and cholinergic neurons, providing potential approaches for Parkinson’s and Alzheimer’s diseases. AAV, adeno-associated virus; Dox, doxycycline; mRNA, messenger RNA; OSK, OCT4/SOX2/KLF4; OSKM, OCT4/SOX2/KLF4/c-MYC; p16, cyclin-dependent kinase inhibitor 2A (p16INK4a); SASP, senescence-associated secretory phenotype.

In vivo, pulsed OSKM activation extended lifespan in progeroid mice, improved multiorgan aging pathology, and increased adult stem cell numbers. In physiologically aged wild-type mice, the same strategy enhanced pancreatic and skeletal muscle regeneration after injury [8]. At the cellular level, OSKM induction reduced senescence-associated gene expression, mitochondrial oxidative stress, senescence-associated β-galactosidase activity, and DNA damage. At the tissue level, it improved age-associated abnormalities in multiple organs, including the skin, spleen, kidney, stomach, vascular system, pancreas, and skeletal muscle [8]. Another study used AAV delivery of OSK factors under the control of the p16 promoter, which is highly active in senescent cells. A single treatment prolonged lifespan and improved multiorgan aging pathology in both progeroid and naturally aged mice, with no increase in tumor incidence observed over the long term [63]. The mRNA expression of the proinflammatory cytokines *Il1a*, *Il1b*, and *Il6* was markedly reduced in multiple organs, with down-regulation of genes involved in inflammatory signaling and up-regulation of genes involved in immune clearance pathways. The accelerated depletion of hematopoietic stem cells observed in LAKI mice was restored, and the cellular frequencies within the hematopoietic stem cell and multipotent progenitor compartments shifted toward those seen in young mice [63]. However, recent in vivo evidence questions whether rejuvenation signatures observed in vitro translate into organismal benefits. Mitchell et al. [121] evaluated a 7-compound partial reprogramming cocktail in middle-aged mice. Low-dose treatment was tolerated but failed to reduce liver or kidney transcriptomic age, whereas higher doses caused weight loss, lipid accumulation, mitochondrial abnormalities, and renal injury without improving rejuvenation. These findings highlight a narrow efficacy–toxicity window and caution against extrapolating in vitro rejuvenation signatures directly to in vivo therapeutic benefits.

Neurodegenerative diseases, represented by PD and Alzheimer’s disease (AD), currently lack effective treatments that slow disease progression due to the nonregenerative nature of neurons and the complexity of their pathology [122]. The main pathological feature of PD is the progressive loss of dopaminergic neurons in the substantia nigra pars compacta. Current treatments primarily rely on dopamine replacement therapy such as levodopa, but efficacy declines over time and is accompanied by severe motor complications [123]. Cell reprogramming technologies offer new possibilities for regenerating functional neurons and reconstructing neural circuits both in vitro and in vivo. In vitro studies relevant to PD have demonstrated the direct conversion of human skin fibroblasts into functional dopaminergic neurons using defined transcription factors [124]. A more clinically relevant in vivo study used Ptbp1 knockdown to induce dopaminergic neuron-like cells from astrocytes. This intervention was associated with partial reconstruction of the nigrostriatal pathway and behavioral improvement in a mouse model of PD [125]. However, this conclusion has been questioned by several independent research groups. Multiple lineage-tracing studies failed to detect newborn neurons after Ptbp1 knockout or knockdown or could not trace these new neurons to astrocytes [126–128]. These studies suggest that regional heterogeneity of astrocytes, AAV dose, and differences in tracing methods may be key factors underlying the conflicting results [129].

For AD, currently approved drugs are mostly symptomatic and cannot reverse or halt the neurodegenerative process, so exploring novel interventions that can regenerate neurons or repair neural networks is urgently needed [130,131]. Sun et al. [44] directly reprogrammed skin fibroblasts into cortical neurons using miRNAs combined with transcription factors, recapitulating key pathological features such as Aβ deposition and tau pathology. Another study used small molecules or transcription factors (e.g., NGN2 and SOX11) to directly convert fibroblasts from healthy individuals and AD patients into functional cholinergic neurons, providing a potential experimental cell source for further evaluation [46,132]. In a mouse model of AD, NEUROD1 treatment was reported to generate neurons attributed to astrocyte-to-neuron conversion and was associated with improved cognitive performance [45]. However, whether these apparent newly generated neurons truly originated from astrocytes has become a major reproducibility concern.

This reproducibility concern has been examined more systematically using rigorous genetic lineage-tracing approaches. Wang et al. [127] systematically reevaluated in vivo astrocyte reprogramming strategies involving NEUROD1, PTBP1, DLX2, and PAX6 using rigorous genetic lineage-tracing systems. The results showed that the so-called newborn neurons previously thought to arise from direct transdifferentiation of astrocytes did not carry astrocyte-derived genetic labels under stringent lineage-tracing conditions and instead mainly originated from preexisting endogenous neurons. This suggests that many previously observed reprogramming events were false-positive signals caused by viral labeling or reporter-system bias rather than genuine cross-lineage fate conversion. The study indicates that claims of highly efficient in vivo transdifferentiation in the direct reprogramming field should be interpreted with great caution. Future studies should use multiple complementary approaches to exclude false-positive reprogramming signals. These include independent genetic lineage tracing, controls in uninjected regions, bromodeoxyuridine prelabeling, detection of immature neuronal stages, and cross-validation of viral and reporter systems. Where possible, in vivo dynamic imaging should provide additional validation [127].

### Regenerative medicine

Regenerative medicine aims to restore damaged tissues through cell replacement, tissue engineering, or activation of endogenous repair. Cell reprogramming technologies, especially direct and partial reprogramming, enable the conversion of easily obtainable somatic cells into functional target cells, thus avoiding the ethical issues, immune rejection, and tumorigenic risks associated with conventional stem cell transplantation. In recent years, this strategy has produced proof-of-concept findings across multiple organ systems, with a limited number of approaches beginning to enter clinical evaluation (Fig. 3).

**Fig. 3.**
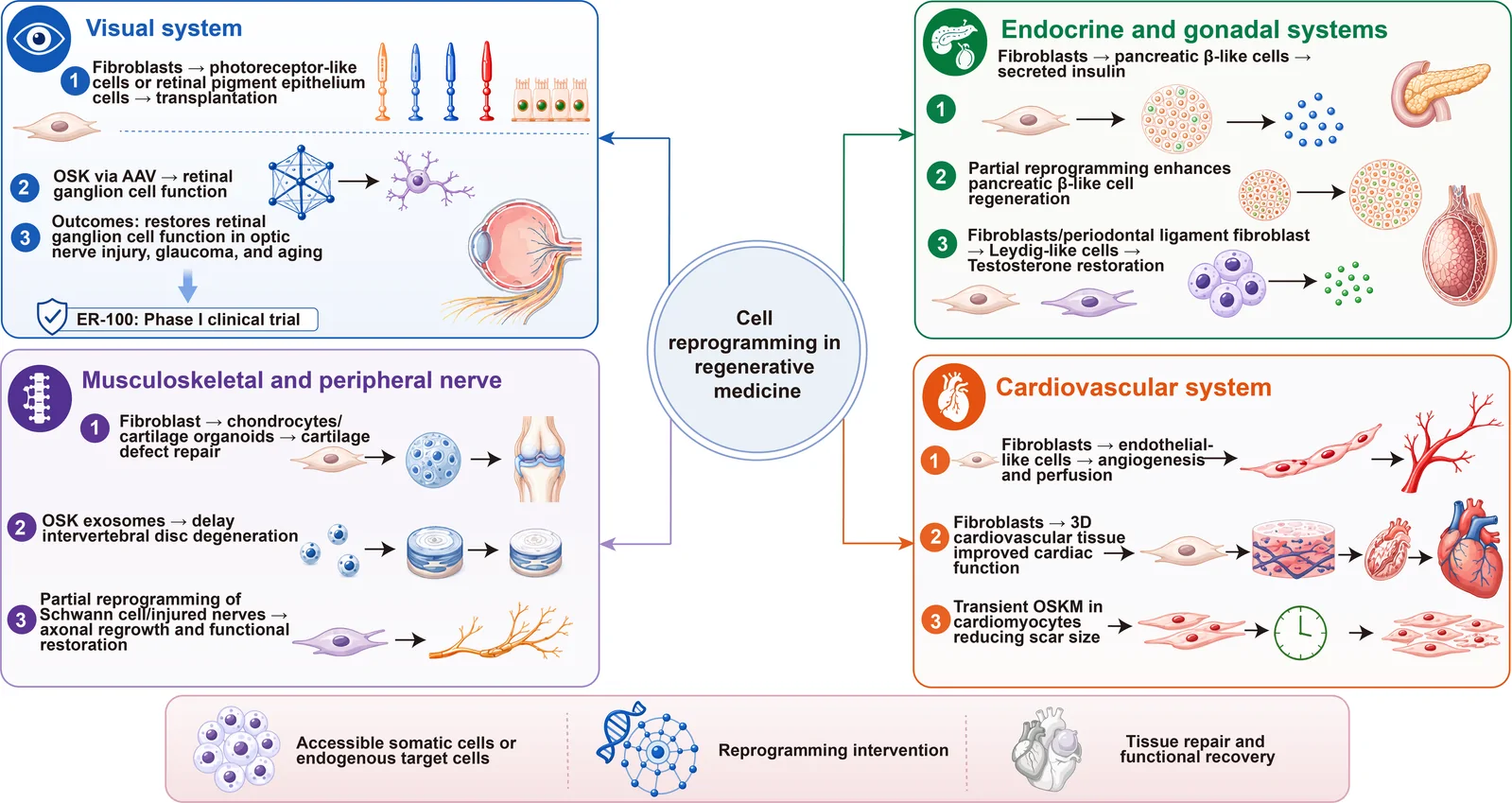
Cell reprogramming strategies in regenerative medicine. Cell reprogramming promotes tissue repair through direct conversion of somatic cells into functional target cells or partial reprogramming of resident cells. Representative applications include generation of photoreceptor-like cells, retinal pigment epithelial cells, pancreatic β-like cells, Leydig-like cells, chondrocytes, endothelial cells, and 3-dimensional cardiovascular tissue. In vivo OSK or OSKM expression and engineered OSK-containing exosomes may further support retinal, pancreatic, intervertebral disc, peripheral nerve, and cardiac repair. ER-100 represents an early clinical-stage application of partial reprogramming for optic neuropathies. 3D, 3-dimensional; AAV, adeno-associated virus; OSK, OCT4/SOX2/KLF4; OSKM, OCT4/SOX2/KLF4/c-MYC.

The visual system represents one of the most advanced fields in this area. At the cell transplantation level, Mahato and colleagues [58] employed chemical small molecules to convert fibroblasts into photoreceptor-like cells; following transplantation into mice with retinal degeneration, some animals exhibited recovery of the pupillary light reflex. Li et al. [133] obtained retinal pigment epithelial cells through chemical reprogramming, which delayed photoreceptor layer degeneration and improved visual function after transplantation. In vivo reprogramming strategies have achieved even more direct effects. For example, Lu et al. [64] delivered OSK factors via AAV targeting mouse retinal ganglion cells, successfully reversing vision loss in models of optic nerve injury, glaucoma, and aging, restoring about 90% of aging-related gene expression to youthful levels. Based on these studies, the first partial reprogramming therapy, ER-100, has received Food and Drug Administration approval to initiate a clinical trial (NCT07290244) for open-angle glaucoma and nonarteritic anterior ischemic optic neuropathy [134]. The ER-100 trial features local ocular AAV-mediated OSK delivery with doxycycline-regulated expression, dose escalation, sentinel participants, independent safety review, and 5-year follow-up. Its design reflects key principles for clinical translation of partial reprogramming: targeting a locally accessible organ, omitting c-MYC, and maintaining terminable expression control. However, the trial has a small sample size, focuses primarily on safety and tolerability, and lacks a control group. Even if visual function improves, functional endpoints alone cannot establish stable epigenetic rejuvenation of human retinal ganglion cells; efficacy and mechanism must be interpreted alongside molecular markers and long-term follow-up [134].

In the endocrine system, Zhu et al. [55] transiently expressed reprogramming factors to induce fibroblasts into pancreatic β-like cells; after transplantation, these cells secreted insulin in response to glucose stimulation and protected diabetic mice from hyperglycemia. Furthermore, in physiologically aged wild-type mice, pulsed activation of OSKM for partial reprogramming followed by low-dose streptozotocin-induced pancreatic β-cell injury resulted in better glucose tolerance recovery and larger islet area in the treated group, indicating that partial reprogramming enhances pancreatic regenerative capacity [8]. In gonadal research, small molecules converted fibroblasts into functional Leydig cells; after transplantation into testosterone-deficient rats, serum testosterone levels returned to near normal and followed a circadian secretion pattern [57]. This technology has also been applied to human periodontal ligament fibroblasts to generate human Leydig-like cells capable of synthesizing testosterone.

In the musculoskeletal system, cell reprogramming has shown therapeutic potential. Chen et al. [56] used small molecules to convert fibroblasts into chondrocytes that formed cartilage organoids; after transplantation, they repaired cartilage defects in mice, restoring approximately 63% of the elastic modulus. In a separate study, exosomes expressing OSK were administered to a rat model of intervertebral disc degeneration, which delayed degenerative processes and relieved pain [65]. In a peripheral nerve injury model, pulsed activation of OSKM at the site of sciatic nerve injury in aged mice promoted axonal regrowth and functional restoration and also reversed Schwann cell senescence [66]. These findings suggest that, beyond directly improving intrinsic cellular states, partial reprogramming may promote tissue repair by remodeling the injury microenvironment and supporting the function of accessory cells.

A recent study generated self-renewable alveolar epithelial-like cells from mouse fibroblasts using NKX2-1, FOXA1, FOXA2, and GATA6 combined with 3D organoid culture [135]. The resulting cells acquired alveolar epithelial type 2-like features and engrafted into injured mouse alveoli. However, conversion efficiency and engraftment remained low, cellular maturation and therapeutic efficacy were limited or unestablished, and the same approach failed in human fibroblasts. These findings highlight that successful lineage conversion in mouse cells may not translate into reproducible or functionally mature human cells.

Beyond incomplete maturation and limited cross-species reproducibility, genuine fate conversion may also be constrained by metabolic mismatch. In a mouse cortical stab-injury model, ASCL1 or NEUROG2 initiated glia-to-neuron transcriptional programs, but most cells died during the conversion window [136]. The study found that activation of the target-lineage transcriptional program outpaced the metabolic transition from glycolysis to oxidative phosphorylation, leading to increased mitochondrial oxidative load, reactive oxygen species, and lipid peroxidation, whereas antioxidant responses such as NRF2 activation were insufficient, ultimately causing ferroptosis-centered cell death [136]. Interventions including BCL-2, Liproxstatin-1, and vitamin E improved cell survival and reprogramming efficiency. These results show that successful direct neuronal reprogramming requires not only establishment of neuronal transcriptional networks but also synchronized metabolic and stress adaptation.

For cardiovascular regeneration, Sayed et al. [137] directly induced human fibroblasts into endothelial cells, markedly improving blood perfusion in a mouse hindlimb ischemia model. Morita et al. [138] found that a single transcription factor, ETV2, is sufficient for this conversion; after transplantation, the cells formed human blood vessels in immunodeficient mice and in a hindlimb ischemia model. Another study used lentiviral transduction of doxycycline-inducible ETV2 together with the transforming growth factor-beta inhibitor SB431542 to further induce expandable endothelial-like cells from human adipose-derived stem cells, also promoting angiogenesis [139]. In cardiac regeneration, Cho et al. [140] reprogrammed fibroblasts into 3-dimensional cardiovascular tissue containing cardiomyocytes; after transplantation, the tissue improved cardiac function. Chen et al. [141] used transient OSKM expression to dedifferentiate and proliferate cardiomyocytes, reducing scar size in a myocardial infarction model; however, long-term expression led to tumor formation, underscoring the importance of spatiotemporal control.

In contrast to these positive cardiovascular advances, the GMT (GATA4/MEF2C/TBX5) combination has encountered bottlenecks in some cardiac reprogramming studies. Although GMT can induce cTnT expression and calcium-channel-related depolarization responses in some mouse fibroblasts, it fails to robustly activate mature cardiomyocyte programs, including *Myh6* (α-MHC) and Nkx2-5 expression. The resulting cells lack spontaneous beating, show limited global transcriptomic change, and exhibit poor survival and phenotype maintenance after in vivo transplantation. This study indicates that expression of a few cardiomyocyte markers or local electrophysiological changes is insufficient to prove completion of mature cardiomyocyte reprogramming from fibroblasts. Subsequent evaluation should cover transcriptional networks, sarcomere structure, calcium handling, spontaneous contraction, electrophysiological maturation, and in vivo functional integration [142]. In addition, applying cell fusion strategies to the cardiomyocyte lineage faces similar obstacles. A study used polyethylene glycol-mediated fusion of cardiomyocytes with embryonic fibroblasts to induce cardiac-lineage reprogramming. The results showed that cardiac gene programs were not effectively activated in most heterokaryons. This failure phenotype suggests that the cardiomyocyte lineage lacks a dominant reprogramming axis comparable to the skeletal muscle MYOD1 axis in fusion systems, indicating that using cell fusion to regenerate cardiomyocytes may require multifactor synergy or higher-level chromatin remodeling [143].

## Challenges in Clinical Translation and Future Directions

The central challenge in cell reprogramming is no longer whether cell fate can be altered, but whether therapeutic reprogramming can be achieved safely, effectively, and controllably. Phenotypic or short-term functional improvements do not necessarily indicate complete or stable fate conversion. Major translational barriers include insufficient reprogramming and delivery efficiency, off-target or abnormal cell-state transitions, tumorigenic risk, and an incompletely defined therapeutic safety window. These challenges also differ between in vivo and ex vivo strategies and therefore require distinct optimization and quality-control frameworks (Fig. 4).

**Fig. 4.**
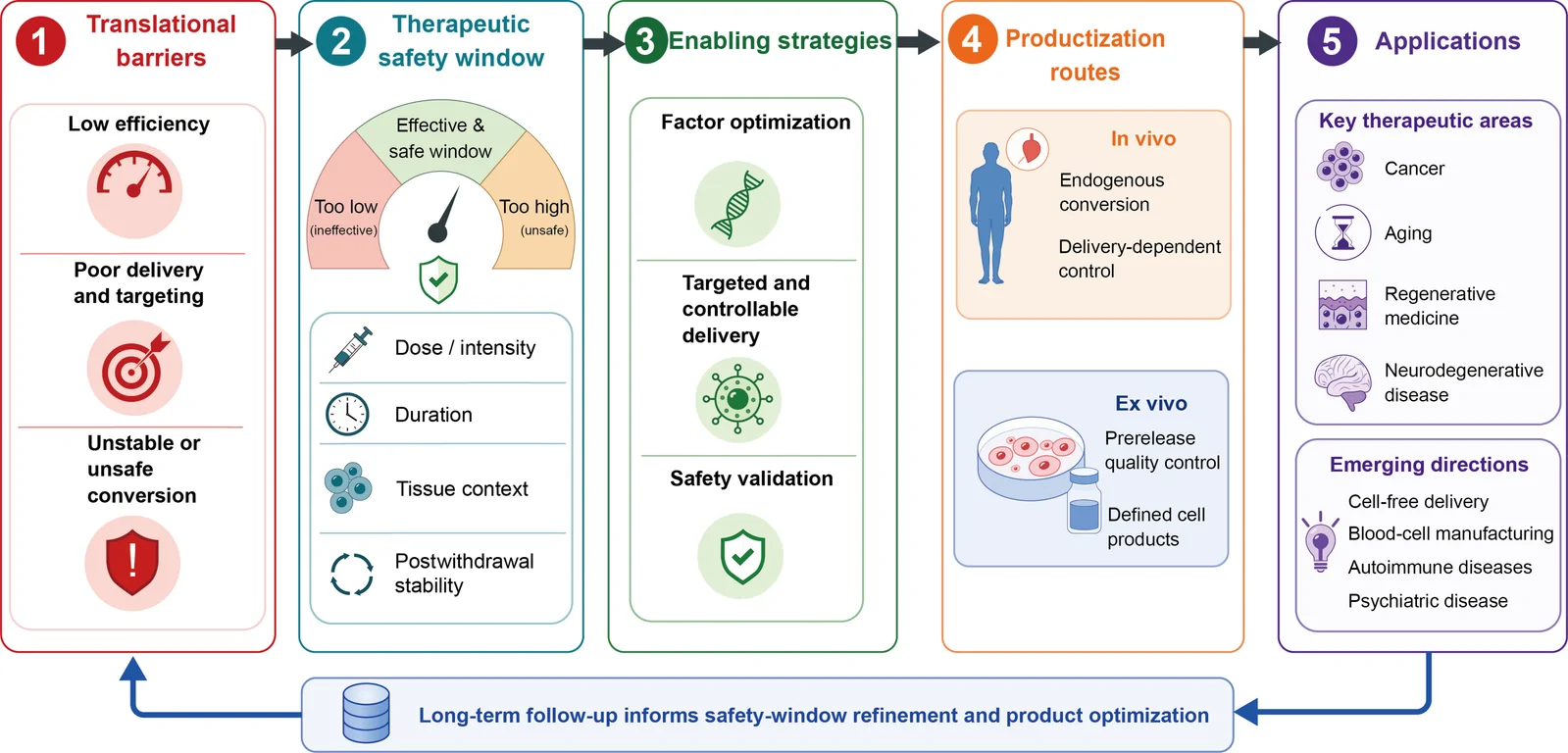
Translational framework for therapeutic cell reprogramming. Major barriers include low efficiency, inadequate delivery and targeting, and unstable or unsafe conversion. Clinical translation requires definition of a therapeutic safety window based on dose, duration, tissue context, and postwithdrawal stability. Enabling strategies include factor optimization, targeted and controllable delivery, and safety validation. Product development should distinguish in vivo from ex vivo reprogramming. These approaches support applications in cancer, aging, regenerative medicine, neurodegenerative diseases, and emerging fields.

### Reprogramming efficiency, fidelity, and therapeutic safety window

Insufficient reprogramming efficiency is the primary barrier limiting the clinical application of cell reprogramming, but its essence is not simply inadequate factor expression. Rather, it reflects the combined effects of starting-cell identity stability and delivery efficiency. Differentiated cells maintain their original identity through repressive chromatin states, lineage-specific enhancer landscapes, DNA methylation patterns, and cell-type-specific gene regulatory networks [5,15]. Recent studies have shown that histone modifications, such as H3K36 methylation, can restrict the access of reprogramming factors to target lineage gene loci, thereby limiting the reprogramming process [144]. The type and state of the starting cell determine reprogramming efficiency. Ideal starting cells should be readily accessible, possess a degree of plasticity, and survive in the target tissue environment. Fibroblasts are currently the most widely used starting cells because they are broadly available, can expand well in vitro, and have developmental plasticity [145]. However, technological advances have expanded the range of starting cells for direct conversion. These include glial cells, adipose-derived stem cells, smooth muscle cells, and pericytes [146]. Cellular senescence further compounds these barriers. Senescent or aged cells are characterized by persistent DNA damage responses, activated p53-p21 and p16-Rb checkpoint pathways, senescence-associated secretory phenotype-associated inflammatory programs and altered chromatin organization [147,148]. These factors may reduce the ability of cells to enter reprogramming trajectories and may increase the probability of cell death or arrest in abnormal intermediate states.

Clinical application is also limited by delivery efficiency and targeting. Viral vectors, lipid nanoparticles, and virus-like particles differ in tissue distribution, cellular uptake, and intracellular release efficiency, and only a limited fraction of reprogramming factors usually reaches target cells and achieves effective expression. Administered dose is not equivalent to effective reprogramming stimulus inside target cells; the fraction of factors that actually reaches target cells and produces sufficient expression intensity and duration is often limited [9]. Imprecise delivery directly leads to poor targeting and risks of off-target reprogramming. After local or systemic administration, reprogramming factors may enter nontarget organs or nontarget cell subpopulations or activate the same signaling pathways outside the intended target cells [9]. This risk is especially important because many reprogramming transcription factors and small molecules regulate broadly expressed epigenetic enzymes or proliferative pathways, and their actions are not naturally restricted to predetermined cells. Beyond the properties of the delivery vehicles themselves, the abnormal physical microenvironment of tissues (e.g., elevated interstitial fluid pressure in tumors) also constitutes an overlooked delivery barrier that may limit the access of systemically administered reprogramming factors to target cells [149]. Although chemical small-molecule reprogramming avoids exogenous gene integration and long-term transgene expression, it still faces problems arising from inconsistent compound combinations, doses, and treatment stages across studies, as well as complex pharmacodynamics, pharmacokinetics, and metabolic toxicity [29].

While improving reprogramming efficiency, defining the boundary between therapeutic benefit and loss of cell identity control is another core issue for clinical translation. This problem is particularly prominent for partial reprogramming. Insufficient stimulation may fail to produce stable functional improvement, whereas excessive or prolonged stimulation may push cells further toward pluripotency or abnormal intermediate states, causing loss of cell identity, disruption of tissue architecture, and tumorigenic risk. Cyclic OSKM induction has been shown to improve aging-associated phenotypes and enhance tissue regenerative capacity in progeroid mice [8]. In physiologically aged mice, long-term cyclic induction for 7 or 10 months produces greater benefits than 1 month of short-term treatment, but reduction in epigenetic age is mainly observed in skin and kidney and is not apparent in several other tissues [150]. This indicates that the effective window for partial reprogramming is strongly tissue specific and cannot be addressed by a uniform dose or induction cycle. In the cardiovascular system, the therapeutic window appears particularly narrow. Short-term OSKM induction can promote cardiac repair, whereas long-term induction causes excessive dedifferentiation and cardiac dysfunction [141]. Therefore, before partial reprogramming enters the clinic, dose-response, time-response, and postwithdrawal stability data must be systematically established across different tissues [151]. Beyond the dedifferentiation risk caused by partial reprogramming, cancer cell reprogramming raises another fundamental safety issue: Phenotypic and lineage changes do not automatically eliminate the original oncogenic driver mutations [9]. Therefore, safety evaluation of cancer cell reprogramming cannot use short-term phenotypic improvement as the sole endpoint. It must also assess whether the original driver mutations persist, whether malignant transcriptional programs are stably suppressed, whether cell states rebound after withdrawal of induction, and whether reprogrammed cells retain long-term clonal expansion or re-tumorigenic capacity. Therapeutic cell fusion requires additional attention to the safety of fusion products and residual tumor cells. Unlike spontaneous fusion in vivo, dendritic cell–tumor cell fusion vaccines are usually prepared ex vivo and irradiated or otherwise treated to inhibit continued proliferation of unfused tumor cells or fusion products [68]. Existing studies in multiple myeloma and other diseases support the feasibility, tolerability, and immunogenicity of this strategy [114,152], but early or medium-scale trials are still insufficient to completely exclude rare delayed risks. Therefore, quality control for fusion vaccines should focus on fusion efficiency, MHC and costimulatory molecule expression, residual proliferative tumor cells, genomic stability, and risks of abnormal clonal expansion or metastasis after reinfusion. For hybrid cells or hybrid cell-derived exosomes, single-cell RNA sequencing, whole-exome sequencing, and monitoring of circulating hybrid cells can help identify their molecular features, immune functions, and potential risks [153–155].

Taken together, the safety window for reprogramming therapy should not be understood as a single dose range, but as a dynamic interval jointly defined by dose, time, tissue context, cell identity stability, and long-term risk [29]. We propose that future studies could consider a prospective GO/NO-GO framework integrating efficacy and safety endpoints [9,60]. For in vivo reprogramming, dose or expression intensity, exposure duration, dosing interval, and treatment cycles could be optimized stepwise to define the minimum biologically effective exposure [8]. GO criteria could require reproducible reprogramming and functional benefit without persistent cell-identity loss, pluripotency activation, abnormal proliferation, tissue injury, or organ dysfunction, whereas the occurrence or persistence of these abnormalities could constitute NO-GO criteria. The minimum effective exposure and the lowest exposure triggering a NO-GO criterion could define the lower and upper boundaries of the therapeutic window, respectively. Regimens within this window could then undergo withdrawal and repeated-cycle testing for delayed or cumulative toxicity. For ex vivo reprogramming, analogous criteria could be incorporated into pretransplantation product release to exclude incompletely reprogrammed, proliferative, or genomically unstable cells [156]. This framework should be validated progressively in human ex vivo tissues and animal models before clinical translation.

### Strategies to improve reprogramming efficiency, targeting, safety, and clinical translation

Improving clinical translation requires coordinated solutions for 3 major challenges: intrinsic cell-identity barriers, insufficient delivery and targeting, and safety risks. These challenges should be addressed together because increasing reprogramming intensity may improve conversion efficiency while simultaneously narrowing the safety margin.

Overcoming epigenetic barriers is the foundation for improving reprogramming efficiency. Transcription factors, miRNAs, and small molecules can be combined to weaken starting-cell identity programs and facilitate lineage conversion [157]. For example, vitamin C can promote DNA and histone demethylation through TET- and JmjC-associated dioxygenases [158]; histone deacetylase inhibitors such as valproic acid can increase the responsiveness of somatic cells to reprogramming factors [159], and miR-9/9-124 can suppress pan-somatic gene networks during neuronal reprogramming [146].

Optimization of factor combinations is equally important. Single-cell multiomics can resolve intermediate states and identify key regulatory nodes and optimal intervention timing [160]. DRUG-seq combined with multimodal multiomics has been used to refine small-molecule combinations [133]. Artificial intelligence virtual cells may further move reprogramming research from descriptive analysis toward predictive design by integrating transcriptomic, epigenomic, and perturbation data to prioritize factor combinations and timing schemes [161]. Early tools include single-cell foundation models such as scGPT [162] and gene regulatory network-based prediction platforms such as CellOracle [163]. However, these models mainly narrow the candidate space; predictions remain sensitive to data quality and coverage and require validation by Perturb-seq, lineage tracing, organoids, and in vivo models [161,164,165]. Evaluation should include not only target-marker expression but also functional maturity, state stability, and abnormal proliferation risk. CRISPR activation screens targeting lineage-determining promoters and enhancers may further help identify regulatory elements that improve conversion without activating pluripotency or proliferative programs [166].

Even an optimized reprogramming program has limited therapeutic value without precise and controllable delivery. AAV provides durable expression and broad tissue tropism but is limited by payload capacity, tissue specificity, immune responses, preexisting neutralizing antibodies, and difficulty with repeated administration. Capsid engineering, cell-specific regulatory elements, and miRNA detargeting may improve selectivity [167]. By contrast, mRNA-LNPs enable flexible, nonintegrating, and transient expression, but tissue targeting, intracellular release, repeated-dose immune responses, and off-target expression remain limitations [168]. At present, transient messenger RNA-loaded lipid nanoparticles and viral vectors such as AAV have not been systematically compared in the context of cell reprogramming with respect to expression duration, tissue distribution, immunogenicity, reprogramming efficiency, and off-target risk. To standardize delivery, we propose a stepwise framework linking product quality, delivery performance, and biological response [169]. First, key product parameters, including composition, loading, potency, stability, and batch consistency, should be controlled. Second, biodistribution, cellular uptake, expression kinetics, and off-target exposure should be quantified, with emphasis on active exposure in target cells rather than administered dose alone. Finally, these parameters should be linked to reprogramming efficiency, functional benefit, and toxicity to define the minimum effective exposure and upper safety limit. This framework could facilitate head-to-head comparisons and selection of tissue-specific strategies.

Successful delivery and fate conversion must also be accompanied by functional maturation and safety control. Three-dimensional culture and tissue engineering can improve tissue organization and integration [140]. For example, chemically converted chondrocytes formed scaffold-free cartilage organoids that promoted cartilage repair and restored mechanical function in vivo [56]. At the same time, improved functional integration must be accompanied by mechanisms to control or terminate potentially harmful cell states. For example, an inducible caspase-9 suicide switch can eliminate cells when severe adverse reactions, abnormal proliferation, or tumor-formation risks occur [170]. In addition, original driver mutations may persist after cancer cell reprogramming. Therefore, future studies could explore combining differentiation or reprogramming therapies with driver-gene inhibition, targeted therapy, or gene editing to reduce the possibility of reactivation of malignant programs.

A further translational decision is whether reprogramming should be performed in vivo or ex vivo. Head-to-head comparisons in the same disease context remain lacking, and neither route is universally superior. The choice should depend on target-organ accessibility, starting-cell availability, requirements for functional integration, safety, quality control, and manufacturing feasibility. The advantage of in vivo reprogramming lies in in situ conversion and potential integration within the tissue. By converting endogenous cells in situ, it may avoid cell loss associated with ex vivo manipulation and transplantation and reduce the need for allogeneic cell replacement [171]. As conversion occurs in the native microenvironment, resulting cells may retain anatomical position and intercellular connections, making this approach suitable for structurally complex or connectivity-dependent organs such as the heart, retina, and central nervous system [171,172]. The main challenge is that final cells cannot be prescreened or released individually; incompletely reprogrammed, abnormal intermediate, or proliferative cells cannot be excluded in advance. Therefore, productizing in vivo reprogramming requires shifting quality control upstream to vectors, active ingredients, and delivery systems, with robust evaluation of tissue distribution, cell selectivity, expression kinetics, potency, immunogenicity, and off-target risk.

Ex vivo reprogramming offers comprehensive pretreatment quality control, allowing assessment of identity, purity, viability, maturity, functional potency, proliferative potential, and genomic stability before reinfusion, with abnormal cells removed prior to dosing [173]. This strategy is preferable when therapy demands defined cellular composition, specified mature cell numbers, tissue-engineered constructs, or strict nontumorigenicity confirmation. Its challenges include complex manufacturing, longer timelines, cell loss, phenotypic drift, batch-to-batch variation, and higher costs. Posttransplantation issues such as immune rejection, poor survival, inadequate vascularization, and suboptimal integration may also arise. Autologous products reduce immune incompatibility but are affected by patient- and starting-cell variability, whereas allogeneic or universal products facilitate cell banking, prerelease testing, and scalable manufacturing but require control of immune compatibility and batch consistency.

The transition from individualized preparation to scalable and productized therapy requires critical quality attributes and release criteria tailored to the mechanism of action. For in vivo reprogramming products, regulatory, clinical trial approval, and productization priorities focus on the delivery system and expression control, ensuring consistency in vector or drug composition, potency, storage stability, tissue selectivity, expression duration, and controllability [174,175]. For ex vivo reprogramming products, the focus is cell identity, purity, functional potency, genetic stability, nontumorigenicity, and batch consistency [173,176]. Different products should also have specific metrics. For example, fusion vaccines should be assessed for fusion rate, MHC and costimulatory molecule expression, antigen-presentation capacity, T-cell activation capacity, and residual tumor cells. Neuronal, cardiomyocyte-like, or endocrine-like cells should be assessed for electrophysiological function, stimulus-responsive secretion, or tissue-integration potential. Future studies should establish comparable efficacy, safety, and manufacturing evaluation systems in specific disease contexts, rather than judging abstractly which route is superior.

### Emerging applications and expanding boundaries

Future applications of cell reprogramming can draw on experience from the iPSC field, particularly in scalable manufacturing, extracellular-vesicle delivery, and disease modeling. Emerging directions include cell-free delivery, blood-cell production, autoimmune disease, and neuropsychiatric disease modeling.

Extracellular vesicles (EVs) represent a potential cell-free platform. iPSC-derived EVs can be engineered for tissue targeting, deliver RNA or protein cargoes, and modulate inflammation and tissue repair [177,178]. EVs generated during cardiomyocyte differentiation have also been used to promote fibroblast-to-cardiomyocyte conversion [179]. However, lineage tracing, single-cell multiomics, withdrawal studies, and long-term functional validation are still needed to distinguish genuine fate conversion from transient paracrine or marker-level effects.

Cell manufacturing is another area that can draw on iPSC experience. iPSC-derived platelets have reached approximately 10^^^11-scale production using expandable megakaryocyte progenitor banks and turbulent bioreactors, with autologous infusion studies providing preliminary evidence of feasibility and safety [180]. Beyond scalable manufacturing, recent Japanese clinical studies also provide a reference for the clinical translation of differentiated cell products. Clinical studies in Japan have extended this experience to iPSC-derived dopaminergic progenitors for PD [181] and cardiomyocyte sheets for cardiomyopathy [182]. Together, these experiences highlight that clinical translation requires not only reproducible cell generation and manufacturing but also transplantation safety, long-term graft survival, functional integration, and robust efficacy validation.

By contrast, direct reprogramming to generate erythroid or megakaryocyte-like cells remains largely at the proof-of-concept stage. Existing studies show that transcription factor-mediated direct reprogramming can convert fibroblasts into erythroid progenitor-like cells or megakaryocyte-like cells [183,184], and cell fusion can also induce partial hematopoietic-like reprogramming. However, the successful fusion and expansion efficiency in that study was only approximately 0.018% [185]. Current evidence does not yet establish the translational feasibility of these strategies. Key unresolved issues include efficient erythrocyte enucleation, stable adult hemoglobin expression, clinical-grade platelet release, effective in vivo infusion, and scalable production. Therefore, the value of direct reprogramming in blood-product manufacturing still requires further validation in terms of functional maturity, production efficiency, infusion safety, and batch manufacturing capacity.

Autoimmune diseases may become an important new application area for cell reprogramming. Immune cells have substantial state plasticity, and pathogenic processes often involve abnormal remodeling of immune-cell identity or functional states. For example, *FOXP3* mutations can cause regulatory T cells to acquire T helper 2 (Th2)-like effector functions, disrupt immune tolerance, and drive autoimmune polyendocrinopathy [186]. This study is not therapeutic reprogramming, but it suggests that key nodes maintaining immune-cell identity stability may become intervention targets. In therapeutic exploration, the small molecule aminooxyacetic acid can reprogram proinflammatory human Th17 cells into induced regulatory T cells by regulating cellular metabolism and epigenetic state [59]. Deletion of *Bcl11b* can transdifferentiate mouse T cells into natural killer-like cells [187], whereas transient *Hoxb5* expression can convert pro-B cells into early T-lineage progenitors [188]. These findings demonstrate immune-cell plasticity but remain distant from therapeutic application. A more clinically relevant direction is to redirect pathogenic Th17 cells, abnormal B cells, or inflammatory myeloid cells toward immunoregulatory states and evaluate their stability, antigen specificity, immunosuppressive risk, and in vivo safety.

Psychiatric disorders and complex neurodevelopmental diseases are more likely to benefit first from reprogramming-mediated disease modeling and drug screening rather than direct cell therapy. Diseases such as schizophrenia, bipolar disorder, and autism involve polygenic risk, abnormal developmental timing, altered neural circuit function, and interactions with environmental factors. Direct replacement of a single cell type is usually insufficient to explain or reverse the full pathological process. Patient-derived iPSC-differentiated neurons, astrocytes, microglia, and brain organoids have been widely used to study disease-associated developmental abnormalities, neural network function, and drug responses [189]. Compared with iPSC models, neurons obtained by direct reprogramming may retain more donor age and some disease-associated epigenetic features, making them more suitable for studying adult-onset or age-related psychiatric phenotypes. Combining such models with organoids, multiomics, and drug screening may help identify disease subtypes, actionable pathways, and individualized drug responses.

## Conclusion

In summary, the essence of cell reprogramming lies in its systemic and holistic perspective, which is highly consistent with the philosophy of integrative medicine. Just as microbiota in different parts of the human body form an integrated ecosystem through signaling interactions with host cells [190], direct reprogramming, partial reprogramming, and cell fusion-induced reprogramming, although following different routes, all depend on global reconstruction of cell-fate networks and epigenetic states. The clinical key is how to exploit plasticity while avoiding loss of identity control and tumorigenic risk. Current translation still faces challenges in efficiency, maturity, safety windows, and genetic stability, especially in cancer contexts, where long-term safety and functional stability must be verified. Future work should shift from simply improving efficiency toward building controllable, reproducible, and verifiable clinical systems, integrating multiomics, lineage tracing, and artificial intelligence-based optimization strategies, and developing safe, regulatable delivery methods and differentiated quality standards. In terms of applications, reprogramming is expanding towards cell-free delivery, blood-cell manufacturing, autoimmune diseases, and psychiatric disease modeling, but its stability and clinical value require confirmation through long-term and multidimensional assessment. Overall, before cell reprogramming enters the stage of clinical translation, a closed loop must be established among mechanistic analysis, safety control, delivery optimization, and quality standards.
