# Supplementary material for: Cell Reprogramming: Therapeutic Advances and Challenges
Source: Research (Wash D C). 2026 Nov 10;9:1472. doi: 10.34133/research.1472 (PMC13748628; doi:10.34133/research.1472)
Supplement: Supplementary file 1 [file research.1472.f1.pdf]

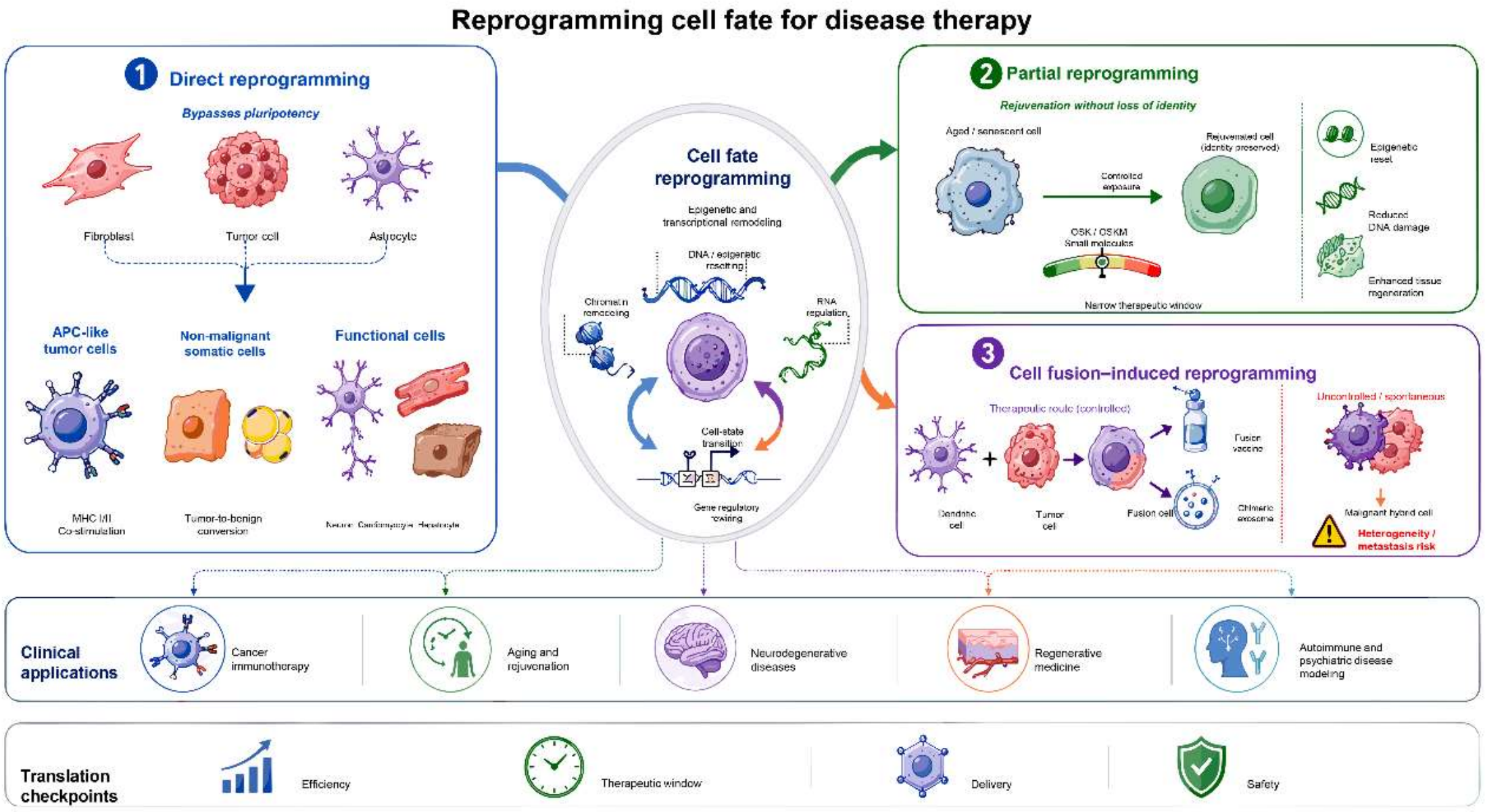


**Graphical Abstract. Reprogramming cell fate for disease therapy.** Direct reprogramming converts somatic or diseased cells into functional target cells while bypassing pluripotency. Partial reprogramming aims to reverse aging-related cellular states while preserving cell identity. Cell fusion-induced reprogramming can be harnessed for antitumor immunity through fusion vaccines and chimeric exosomes, although uncontrolled fusion may also generate malignant hybrid cells. These strategies provide potential therapeutic routes for cancer immunotherapy, aging and rejuvenation, neurodegenerative diseases, regenerative medicine, autoimmune diseases, and psychiatric disease modeling. Successful translation will depend on improving reprogramming efficiency, defining safe therapeutic windows, optimizing delivery systems, and ensuring long-term safety.
